# Person-Centered Care in the Home Setting for Parkinson's Disease: Operation House Call Quality of Care Pilot Study

**DOI:** 10.1155/2015/639494

**Published:** 2015-05-19

**Authors:** Nawaz Hack, Umer Akbar, Erin H. Monari, Amanda Eilers, Amanda Thompson-Avila, Nelson H. Hwynn, Ashok Sriram, Ihtsham Haq, Angela Hardwick, Irene A. Malaty, Michael S. Okun

**Affiliations:** ^1^University of Florida Health Center for Movement Disorders and Neurorestoration, McKnight Brain Institute, University of Florida, Gainesville, FL, USA; ^2^Alpert Medical School, Brown University, Providence, RI, USA; ^3^Florida Neurology Group, Ft Meyers, FL, USA; ^4^Division of Neurology, Scripps Clinic Torrey Pines, San Diego, CA, USA; ^5^Spectrum Health Medical Group, Michigan State University, Grand Rapids, MI, USA; ^6^Department of Neurology, Wake Forest School of Medicine, Winston-Salem, NC, USA; ^7^Norton Healthcare, Louisville, KY, USA

## Abstract

*Objective*. (1) To evaluate the feasibility of implementing and evaluating a home visit program for persons with Parkinson's disease (PD) in a rural setting. (2) To have movement disorders fellows coordinate and manage health care delivery. *Background*. The University of Florida, Center for Movement Disorders and Neurorestoration established Operation House Call to serve patients with PD who could not otherwise afford to travel to an expert center or to pay for medical care. PD is known to lead to significant disability, frequent hospitalization, early nursing home placement, and morbidity. *Methods*. This was designed as a quality improvement project. Movement disorders fellows travelled to the home(s) of underserved PD patients and coordinated their clinical care. The diagnosis of Parkinson's disease was confirmed using standardized criteria, and the Unified Parkinson's Disease Rating Scale was performed and best treatment practices were delivered. *Results*. All seven patients have been followed up longitudinally every 3 to 6 months in the home setting, and they remain functional and independent. None of the patients have been hospitalized for PD related complications. Each patient has a new updatable electronic medical record. All Operation House Call cases are presented during video rounds for the interdisciplinary PD team to make recommendations for care (neurology, neurosurgery, neuropsychology, psychiatry, physical therapy, occupational therapy, speech therapy, and social work). One Operation House Call patient has successfully received deep brain stimulation (DBS). *Conclusion*. This program is a pilot program that has demonstrated that it is possible to provide person-centered care in the home setting for PD patients. This program could provide a proof of concept for the construction of a larger visiting physician or nurse program.

## 1. Introduction

Parkinson's disease (PD) remains a major cause of disability worldwide. There are an estimated one million people diagnosed with PD in the United States (US). It has been estimated that $6.3 billion dollars are spent in indirect costs, such as missed work for a patient or family member. There is a need for early diagnosis of PD and a review by specialty-trained neurologists to facilitate early access to proper health care resources and to early interventions [1]. There are also important travel related expenses for many families seeking expertise from a neurologist or a movement disorder specialist [1].

PD patients spent 1.9 million U.S. hospital inpatient days in 2010 for PD related causes [1]. Inpatient days were 73% higher when compared to populations free of PD [1]. PD patients required more specialized medical care and more regularly scheduled visits, presumably because of the complexity of treating the motor and nonmotor aspects of the syndrome. As the population ages, the PD burden will continue to grow [2]. There is a critical unmet need for programs that will allow PD patients to remain in home, provide person-centered care, and curb unnecessary hospitalization [3].

Though telemedicine has been recently introduced to select PD populations, there is a paucity of information on the utility of home-based visits in PD [4]. Telemedicine has shown some efficacy in the care of patients with PD, but not every client has access to a telephone or to Internet as was present in our cohort.

We present a fellow initiated quality improvement project (QIP) of a home-based program based in the state of Florida. Our aim was to provide access to health care for patients with PD in our community who could not afford transportation or the cost of an office visit, and we also aimed to evaluate the effectiveness of our intervention. The study was designed to provide a true person-centered model of care. The person-centered model in our case involved movement disorders fellows and a clinical coordinator visiting the home of each patient. At the visits, there was a discussion of individual symptoms and a definition was constructed of the person's goals for care and treatment. The intervention was individually tailored to achieve personal goals.

The program, Operation House Call (OHC), was implemented to fill a gap for PD patients who could not afford to travel or for those without health insurance. We aimed (short- and long-term) to develop a proof of principle that we could improve quality of life, reduce nursing home placements, and reduce hospitalization due related to PD complications. We present our data from this quality improvement person-centered care project.

## 2. Methodology

This project was a clinical quality improvement initiative implemented to improve the access to health care for persons with PD living in the state of Florida. Permission for a retrospective chart review was obtained by the University of Florida institutional review board. The program review covered the period from January 2011 to January 2014.

### 2.1. Inclusion Criteria

The inclusion criteria for participation in the OHC program were a presumed diagnosis of PD, along with an inability to access the health care system usually as a result of the cost of transportation or due to insurance issues. There were no exclusions based on the severity of disease, preexisting medical conditions, psychosis, or dementia.

### 2.2. Exclusion Criteria

Patients with the ability to afford transportation and pay for office visits were excluded.

## 3. Implementation of OHC

To implement OHC, a funding source had to be secured. In our case a private foundation was able to support us and we were able to secure support from the university for time and for using the electronic medical record for our patients. Once we were able to secure funding and the electronic medical record, the project proceeded. Fellows and the clinical coordinator were able to arrange coverage for their absence in both the university clinic and in the hospital during the OHC visit.

The quality improvement project followed up 7 patients at 3–6-month intervals. Each visit interaction was a true home visit performed by a neurology trained movement disorders fellow along with a program coordinator. The transportation was provided by private vehicle, but the Center for Movement Disorders reimbursed the cost of gas. Each visit was videotaped and appointments were scheduled in advance so family members could be present. The person's medical record was reviewed before each visit. Each visit consisted of a history and physical examination, and also interviews with the family members and caregivers.

## 4. Evaluation of OHC

All visits were videotaped and later presented during a weekly interdisciplinary care conference. These conferences included participation from speech pathology, physical therapy, occupational therapy, neuropsychology, psychiatry, neurosurgery, and movement disorders neurology. Each visit was captured in the electronic medical record (EPIC). There was complete documentation for the history and physical examination, the Unified Parkinson's Disease Rating Scale motor part III (UPDRS), and a clinician global improvement survey (CGIS). There was also continuous monitoring of patient retention, frequency of visits to the emergency room, hospitalization due to PD complications, UPDRS motor scales, and clinician global impression (CGIS) scores (all clinically collected for best care).

### 4.1. Data Analysis

Wilcoxon signed-rank tests using SPSS 22.0 were performed to evaluate whether participants significantly improved on UPDRS score and Hoehn and Yahr stage after their Operation House Call participation. Demographic data (such as participant age and distance from center) was reported using mean and standard deviation.

## 5. Results

There were a total of seven patients who had home visits that were initiated from January 2011 to January 2014. One patient was diagnosed with a psychogenic movement disorder and was provided with counseling therapy and later was discharged. The sixth patient (see Table 1) was able to obtain medical insurance after 4 visits (one year of OHC) and later was successfully implanted with a deep brain stimulator and discharged from the program with follow-up in the clinic setting. Five patients remain active in OHC.

There were 3 males and 3 females in the cohort with PD, and the mean age was 64 ± 8.2 (SD). Five patients were initially enrolled due to inability to obtain insurance, and one patient was unable to afford the cost of transportation. All of the patients who could not afford insurance cited a hardship in affording the transportation necessary to see a medical specialist. The cohort reported latency to being evaluated by a neurologist of 1.4 years (mean) (range .025 to 2 years). The reasons included lack of access to a local neurologist (*n* = 3) and financial burden (*n* = 6).

The medication regimens prior to enrollment in OHC are summarized in Table 1. Briefly, *n* = 3 patients were not on PD medical therapy and *n* = 2 patients were prescribed carbidopa/levodopa by a local general practitioner but had not initiated the medication. The reason cited by both patients was a concern about the accuracy of the diagnosis. One patient started amantadine 6 months prior to OHC. Following OHC *n* = 6 patients were prescribed carbidopa/levodopa; *n* = 1 patient was prescribed citalopram for depression; and *n* = 1 patient was prescribed escitalopram also for the indication of depression.

CGIS scores were much improved (a rating of 2/7) for all six patients. A clinical coordinator checked in regularly with patients by phone to ensure adherence to the new medication regimens. Two patients were identified with clinically significant depression without suicidal ideation, and both were treated without the need for hospitalization. Each patient had a mean of 3.67 ± 0.52 (SD) home visits a year (Table 1).

Using SPSS 22.0, a Wilcoxon signed-rank test was run to examine whether the UPDRS motor score significantly differed before and after Operation House Call participation. Results revealed significant improvement, *Z* = −2.00, *P* < .05, such that the median score before participation (median = 37; interquartile range 33–47.75) was higher (worse) than the median score following enrollment (median = 23.50; interquartile range 22.75–38.25), 36.5% improvement.

A Wilcoxon signed-rank test was also performed to examine whether the “on” PD medication Hoehn and Yahr stage differed before and after OHC participation. The results revealed no significant median difference in Hoehn and Yahr stage, though the result neared significance (*Z* = −1.89, *P* = .06). Because the dataset contains an even number of cases, there is no true meaningful median (see Table 1 for listing of scores). Averaging the two middlemost scores yields a number that is not meaningful on the Hoehn and Yahr scale; therefore, the two middlemost scores are reported here instead of the true median. Similarly, to present more meaningful results, intertertile ranges are reported rather than interquartile ranges because the dataset of six cases can be evenly divided into tertiles. The two middlemost Hoehn and Yahr scores were 2.5 and 3 prior to OHC, with stages ranging from 2.5 to 5 (intertertile range was 2.5–3). After Operation House Call, the two middlemost Hoehn and Yahr scores were 2 and 2.5, with stages ranging from 2 to 5 (intertertile range was 2–2.5).

Across all OHC patients there were no emergency room visits or hospitalization following initiation of home visits. One patient had an emergency room visit for a fall and required inpatient rehabilitation a week prior to OHC initiation. The cost of transportation for the visits over the course of a year was $526.43/patient, which included the cost of gasoline, the paid time for the coordinator (on the visit), and the wages paid for the neurology fellow during clinic hours. The cost of transportation was paid for by the university funds and private grants and was applied to the centers cost. Patients did not incur any costs for participation in this program. However, the cost of multidisciplinary team participation and the cost of not having a fellow and program coordinator present during clinic hours could not be easily estimated and were therefore not accounted. The mean distance travelled was 56.3 miles with a range of 25.7 to 152.8 miles.

## 6. Discussion

Our pilot person-centered quality improvement project had a small number of participants and though successful, the small numbers were the main limitation. OHC was a true person-centered healthcare intervention. The goal was to improve quality of life for each patient and to individually address and adhere to health care goals and wishes for intervention on a patient-by-patient basis. Movement disorder fellows and also the clinical care coordinator were able to capture in real-time the favorable and unfavorable circumstances impacting care. OHC has been able to deliver care to seven patients and to assist three patients in achieving healthcare independence (insurance and travel). The non-PD psychogenic movement disorder patient was grateful for the diagnosis and was relieved he did not have a neurodegenerative disease. One patient was able to obtain insurance and receive DBS and was successfully reabsorbed into an office-based practice. Across the cohort, there was an improvement in PD motor scores and the global impression scale. Importantly, no patients were hospitalized or visited the emergency room (ER). Recently it was reported that there was greater than a 30% chance per year of an ER visit or hospitalization for those suffering from PD. Although our home based person-centered visit population sample size was small, demonstration of reduced hospitalization could represent important future savings to the healthcare system [3].

In the United States, neurologist care has been demonstrated to make a difference in health care outcomes [5]. In our cohort, there was an average delay of 1.4 years for accessing a neurologist, and this could prove important to health outcomes in PD. A waiting period of 1.4 years for accessing a neurologist would be considered by most experts to be out of the range of accepted standards of care for patients with PD [6]. The reasons for access difficulties were mainly due to the lack of adequate health insurance, inability to access transportation, and the actual cost of transport. Forty-two percent of Medicare beneficiaries in a recent study did not have access to a general neurologist [4–9]. Lack of access has been reported to result in an increased risk of hip fractures, morbidity, and nursing home placement [5]. Studies have also demonstrated increased mortality associated with complications from PD, even within the first six years of diagnosis [9–15]. In our cohort, all PD patients reported one or more issues with access to care. Once OHC was launched, all of the patients were able to establish care through the home visit process. This process led to an improvement in motor functioning as documented by a decrease in UPDRS scores and an improved satisfaction as shown by the CGIS ratings. In future large-scale studies, pre-post measures of quality of life could better evaluate the impact of such programs. Our pilot project was not large enough to deliver economic and other conclusions about this patient population, and this could be left for future study.

Preventative home visits for the elderly have been shown to decrease morbidity and increase satisfaction [16]. Although home visits have been published in several other chronic diseases, in the case of PD it is still a relatively unexplored phenomenon [17, 18]. Other nonoffice based visit programs, such as telemedicine, have been shown to reduce hospitalization and to reduce health care utilization [19]. Virtual home visits using the Internet have also been studied and have been recently shown to be feasible [4]. Several studies have also revealed the feasibility of PD telemedicine, but results have yet to show long-term viability and also access for families without an Internet connection or appropriate computer hardware [20, 21]. These above-mentioned studies had a greater number of participants and cannot be easily compared at this time to our pilot project. We acknowledge this as an important limitation. In our cohort, three participants (42.9%) did not have Internet or telephone access, and home visits were the only viable option.

In summary, the steps to implement the OHC program involved the following:

1. establishing the goal of true person-centered care and providing access to health care for those in need,
2. creating an action plan to implement a timeline to accomplish setting-up and maintaining the program,
3. exploring local sources of funding as well as securing volunteer commitments from multidisciplinary experts and from movement disorders fellows,
4. meeting with the university administration and members of the community to discuss implementation of the project and also establishing access to the existing electronic medical record,
5. recruiting qualified persons with PD and initiating home visits,
6. implementing evaluations of effectiveness of the program through measures such as the UPDRS, hospital emergency visits, and CGIS,
7. presenting cases to an interdisciplinary team in an open video and discussion format.

Barriers to replicating this program include financial cost (travel and specialist time) and availability of qualified personnel. The current project (OHC) was funded through philanthropy, and that approach may not be feasible for expanding future home visit type programs. Another barrier will be obtaining an institutional commitment to allow medical records to be generated and maintained. Additionally, telemedicine will be able to reach some but not all of the patients in need. Finally, future studies will need to explore the detailed economic benefits of a PD Operation House Call program and will need to factor in the cost of having a movement disorders fellow and also a multidisciplinary team involved. Additionally, other intangible factors such as loss of time from a clinic or hospital based practice must be more carefully accounted.

These types of home-based programs may have the potential to reduce nursing home placement, morbidity, mortality, and hospitalization and possibly improve motor and quality of life; however, there is an important need for larger prospective studies in the home setting. The development of person-centered outcome metrics will aid in motivating government sources and insurance carriers to reimburse home care in PD. The OHC program, though small, has been beneficial to our local PD community in providing true person-centered access to care for those who could not otherwise access services. The future viability of this model of patient care will have to be tested with a much larger population.

## Figures and Tables

**Table 1:** Summary of Operation House Call demographics, UPDRS, H & Y scores, and medications before and one year after completion and summery of mood and hospitalization data.

Patient	Age	Disease Duration	Sex	H & Y scores before completion	H & Y one year after completion	UPDRS		Mood disorder	Visits per year	CGIS	Hospitalization	ER visits	Time to Appointment with a neurologist (years)	Medical regimen before OHC	Medical regimen after OHC	Years in OHC	Distance traveled (mi)	Reason in OHC
before completion	one year after completion

1	72

2	62

3	75

4	63

5	53

6	59

Mean (SD)	64 (8.2)

H & Y: Hoehn and Yahr scores; UPDRS: Unified Parkinson's Disease Rating Scale; CGIS: Clinical Global Impression Scale; OHC: Operation House Call.
